# Deep TCR repertoire sequencing reveals relative change in peanut specific clonotype in subjects undergoing rush oral immunotherapy

**DOI:** 10.1186/1710-1492-10-S2-A53

**Published:** 2014-12-18

**Authors:** Philippe Bégin, Kari C Nadeau

**Affiliations:** 1 Department of Pediatrics, Stanford University, Stanford, California, 94305-5208, USA; 2 Department of Medicine, University of Montreal, Montreal, Quebec, H3A 1A1, Canada

## Background

Oral immunotherapy is an emerging therapy currently under investigation for the treatment of food allergy [1]. Underlying mechanisms are thought to involve a switch in the food specific T cell response from Th2 to either Th1, Tr1 and/or Treg. It is unknown whether this change in response results from re-education of existing pathological food-specific T cells or from their replacement by new healthy T cells (change of guard hypothesis).

## Methods

The objective was to evaluate the clonal distribution of peanut specific T cell in subjects with peanut allergy and follow changes in clonotype with treatment using a high-throughput T cell receptor (TCR) sequencing platform. Peripheral blood mononuclear cells (PBMCs) from three subjects undergoing rush oral immunotherapy in a previous trial [2] and three control subjects on avoidance diet were cultured with peanut extract at baseline and at 9 and 18 months. Carboxyfluorescein succinimidyl ester (CFSE)-low peanut proliferating T cells were then isolated by fluorescence-activated cell sorting (FACS) and TCR analysis was performed.

## Results

The CFSE-low proliferating fraction was found to be comprised of between 2000 and 12,000 different T cell clones. However, only between 15 and 25% of proliferating T cells (from 100-400 different clones) were consistently found at all three time points and probably represented true peanut-specific T cells. While the relative frequency of these peanut-specific clones was stable over time in subjects on avoidance diet (R=0.633 to 0.760), it was found to change in subjects undergoing oral immunotherapy (R= 0.123 to 0.350), following two characteristic patterns.

## Conclusions

Using a deep TCR sequencing platform, we found that only a fraction of CFSE-low peanut proliferating T cells were consistent in time and likely to represent true peanut specific T cells. Oral immunotherapy was associated with changes in relative frequency of clones within this fraction, which would support the change of guard hypothesis.
